# Comparative chloroplast genomes of *Dactylicapnos* species: insights into phylogenetic relationships

**DOI:** 10.1186/s12870-024-04989-7

**Published:** 2024-04-29

**Authors:** Shunquan Yang, Juntong Chen, Zhimin Li, Xianhan Huang, Xu Zhang, Qun Liu, Komiljon Tojibaev, Hang Sun, Tao Deng

**Affiliations:** 1 State Key Laboratory of Plant Diversity and Specialty Crops, Kunming Institute of Botany, Chinese Academy of Sciences, Kunming, 650201 China; 2 School of Life Sciences, Yunnan Normal University, Kunming, 650500 China; 3 CAS Key Laboratory of Plant Germplasm Enhancement and Specialty Agriculture, Wuhan Botanical Garden, Chinese Academy of Sciences, Wuhan, 430074 China; 4 Institute of Botany, Academy Sciences of Uzbekistan, Tashkent, 100125 Uzbekistan

**Keywords:** *Dactylicapnos*, Chloroplast genome, Comparative analysis, Phylogeny, Papaveraceae

## Abstract

**Background:**

*Dactylicapnos* is a climbing herbaceous vine, distributed from the Himalayas to southwestern China, and some of the species have important medicinal values. However, the chloroplast genomes of *Dactylicapnos* have never been investigated. In this study, chloroplast genomes of seven *Dactylicapnos* species covering all three sections and one informal group of *Dactylicapnos* were sequenced and assembled, and the detailed comparative analyses of the chloroplast genome structure were provided for the first time.

**Results:**

The results showed that the chloroplast genomes of *Dactylicapnos* have a typical quadripartite structure with lengths from 172,344 bp to 176,370 bp, encoding a total of 133–140 genes, containing 88–94 protein-coding genes, 8 rRNAs and 37–39 tRNAs. 31 codons were identified as relative synonymous codon usage values greater than one in the chloroplast genome of *Dactylicapnos* genus based on 80 protein-coding genes. The results of the phylogenetic analysis showed that seven *Dactylicapnos* species can be divided into three main categories. Phylogenetic analysis revealed that seven species form three major clades which should be treated as three sections.

**Conclusions:**

This study provides the initial report of the chloroplast genomes of *Dactylicapnos*, their structural variation, comparative genomic and phylogenetic analysis for the first time. The results provide important genetic information for development of medical resources, species identification, infrageneric classification and diversification of *Dactylicapnos*.

## Introduction

*Dactylicapnos* Wall. belongs to Fumarioideae (DC.) Endlicher. in the family Papaveraceae Juss., established by Wallich in 1826 [1]. There are about 15 species in the genus, distributed from the Himalayas to southwestern China [2–5]. *Dactylicapnos* is a climbing herbaceous vine, distinguished by its branched tendrils at the end of leaves, pendent raceme inflorescences, yellow bisymmetric flowers and subquadrangular stigma with a papilla on each corner [2]. Taxa of *Dactylicapnos* are rich in active ingredients such as isoquinoline alkaloids [6], with the highest content of isocorydine and protopine [7], and is used in a Bai Nationality folk medicine due to its analgestic, anti-inflammatory, hemostatic and anti-hypertensive effects [8].

Despite some species of *Dactylicapnos* are very important medicinal plants, the relationship between these species is not clear. Recent classification of *Dactylicapnos* by Lidén and Pathak [5] based on morphology divided the genus into three sections (sect. *Dactylicapnos*, sect. *Minicalcara* and sect. *Pogonosperma*) and two informal groups. Validity of these morphologically defined sections and informal groups could not be confirmed due to lack of a systematic molecular study of the genus. A few molecular studies performed to date included only a few species of the genus, Lidén [9] used *rps16* fragments to explore the systematic relationship between *Dicentra* and *Dactylicapnos*, Pérez-Gutiérrez [10, 11] conducted a molecular phylogenetic study of the Fumarioideae using five plastid markers, which included only four *Dactylicapnos* species, and only three species were included in the study by Chen [12]. There has never been a systematic molecular study to resolve the genus infrageneric phylogeny based on cp genome. Thus, the monophyly of these morphologically defined sections and informal groups could not be confirmed to verify the Lidén and Pathak [5] classification.

Chloroplasts are important organelles for photosynthesis in green plants which genome uniparently inherited. The chloroplast (cp) genome size of angiosperms is in the range of 120 to 160 kb [13], with a typical quadripartite structure, consisting of two-copy inverted repeat (IR) of 20–28 kb, a large single-copy regon (LSC) of about 80–90 kb and a small single-copy region of 16–27 kb [14], usually encoding for 120–150 genes. It is known that the cp genome encodes all the tRNA and rRNA molecules and partial proteins required for its own function [15–17]. Due to the highly conservative structure, rich in genetic information [18], slow nucleotide substitution rate [19], and uniparental inheritance [20], the cp genome has been widely used in phylogenetics analyses and identifications [21]. At present, chloroplast genome sequences of many species have been published, but the species of *Dactylicapnos* has not been published yet. The lack of systematic molecular studies hampers the development and application of *Dactylicapnos*. This motivated the current study of a comparative genomic analysis of seven of *Dactylicapnos* species covering all three sections and one informal group, in order to understanding the evolution of the genus structure and clarification of the phylogenetic relationship in *Dactylicapnos* species.

## Results

### Chloroplast genome structure and characteristics analyses of *Dactylicapnos* species

**Fig. 1:**
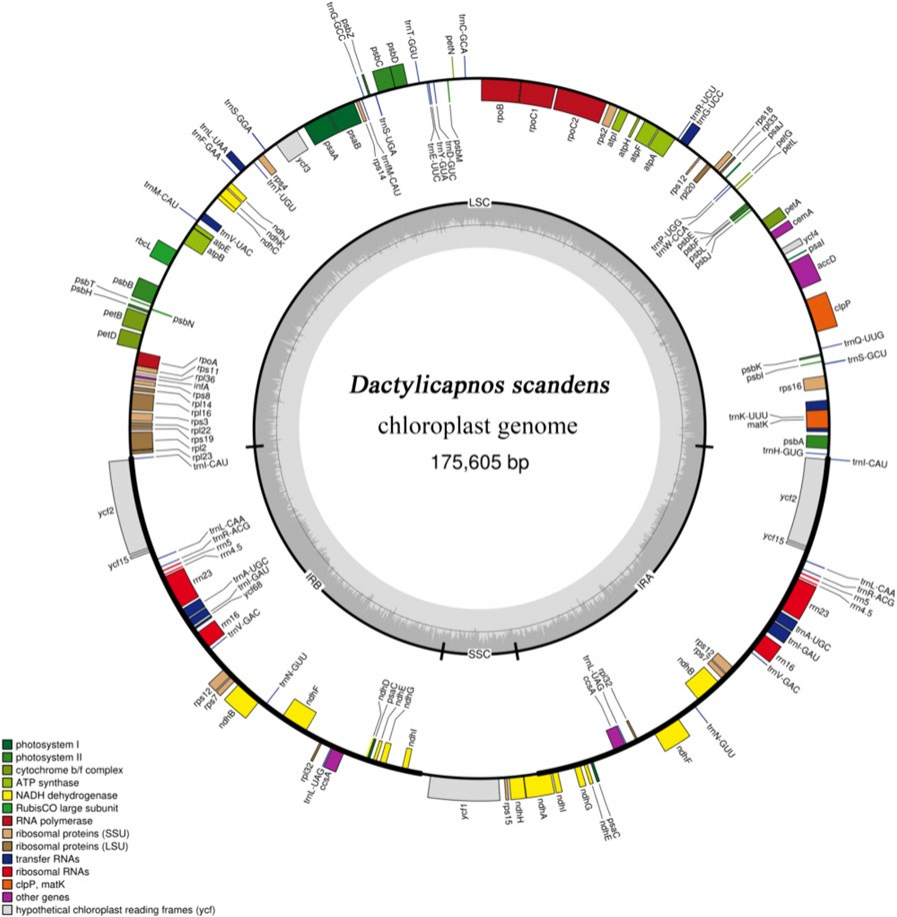
Gene map of the chloroplast genomes of *D. scandens*. Genes inside the circle are transcribed clockwise, and those on the outside are transcribed counter-clockwise. Genes belonging to different functional groups have been color-coded. The darker grey area in the inner circle corresponds to GC content, while the lighter grey corresponds to AT content

The lengths of the studied cp genomes varied from 172,344 bp (*Dactylicapnos schneideri* (Fedde) Lidén.) to 176,370 bp (*Dactylicapnos grandifoliolata* Merrill.), with a typical quadripartite structure, a pair of IR regions (28,530 bp–37,115 bp), LSC regions (89,195 bp–101,092 bp) and SSC (9303 bp–26,089 bp) (Fig. 1; Table 1). In the studies species there was an identical level of GC content with the total content 40.0%–40.6%, 41.6%–43.6% in IR, 39.1%–39.4% in LSC, and 35.3%–38.0% in SSC. The GC content of IR region was higher than LSC and SSC.

**Table 1:** The basic chloroplast genome information of seven *Dactylicapnos* species

Characteristics	*D*. *macrocapnos*	*D*. *scandens*	*D*. *schneideri*	*D*. *grandifoliolata*	*D*. *torulosa*	*D. lichiangensis*	*D. roylei*
Total length(bp)	175,552	175,605	172,344	176,370	174,101	175,134	173,878
LSC lenghth(bp)	92,019	92,352	89,195	90,735	91,031	91,348	101,092
IR lenghth(bp)	37,115	37,018	28,530	36,484	28,922	28,937	28,921
SSC lenghth(bp)	9303	9217	26,089	12,667	25,226	25,912	14,944
Total numble of genes	140	140	139	139	133	133	133
Protein-coding genes	94	94	92	92	88	88	88
tRNA genes	38	38	39	39	37	37	37
rRAN genes	8	8	8	8	8	8	8
Overall GC content(%)	40.0%	40.0%	40.2%	40.2%	40.6%	40.6%	40.6%
GC content in LSC(%)	39.1%	39.1%	39.1%	39.1%	39.4%	39.4%	39.3%
GC content in IR(%)	41.6%	41.7%	43.5%	42.0%	43.6%	43.6%	43.6%
GC content in SSC(%)	35.3%	35.4%	36.9%	36.5%	38.0%	38.0%	37.3%

The seven cp genomes have 133–140 genes, including 88–94 protein-coding genes, 37–39 tRNA genes, and 8 rRNA genes (Table 2). In the studied species, there are 18–26 genes with two copies, which were mostly comprised of seven protein-coding genes (*ycf2*, *ycf15, ycf68*, *rps12*, *rps7*, *ndhB*, *ndhF*), seven tRNA genes (*trnI-CAU*, *trnL-CAA*, *trnR-AGC*, *trnA-UGC*, *trnI-GAU*, *trnV-GAC*, *trnN-GUU*), and four rRNA (*rrn5*, *rrn4.5*, *rrn23*, *rrn16*), but the *D. grandifoliolata* also has six protein-coding genes (*rpl32*, *ccsA*, *ndhD*, *psaC*, *ndhE*, *ndhG*), two tRNA genes (*trnH-GUG*, *trnL-UAG*), *D. schneideri* also has one tRNA gene (*trnH-GUG*), and *Dactylicapnos scandens* Hutch. also have seven protein-coding genes (*rpl32*, *ccsA*, *ndhD*, *psaC*, *ndhE*, *ndhG*, *ndhI*) and one tRNA gene *(trnL-UAG*). Sixteen genes (*trnG-UCC*, *atpF*, *rpoC1*, *trnL-UAA*, *trnV-UAC*, *petB*, *petD*, *rpl16*, *rpl2*, *trnA-UGC*, *trnI-GAU*, *rps12*, *ndhB*, *ndhA*, *trnK-UUU*, *rps16*) contain a single intron, two genes (*ycf3*, *clpP*) have two introns, and the gene *trnK-UUU* has the largest intron, which contains the *matK* gene.

**Table 2:** The basic chloroplast genome information of seven *Dactylicapnos* species

Category of genes	Group of genes	Name of genes
Photosynthesis related genes	ATP synthase	*atpA*,*atpB*,*atpE*,atpF*,*atpH*,*atpI*
	NADH-dehydrogenase	ndhA*,ndhB*,*ndhC*,*ndhD*,*ndhE*,*ndhF*,*ndhG*,*ndhH*,*ndhI*,*ndhJ*,*ndhK*
	Cytochrome b/f complex	*petA*,petB*,petD*,*petG*,*petL*,*petN*
	Photosystem I	*psaA*,*psaB*,*psaC*,*psaI*,*psaJ*
	Photosystem II	*psbA*,*psbB*,*psbC*,*psbD*,*psbE*,*psbF*,*psbH*,*psbI*,*psbJ*,*psbK*,*psbL*,*psbM*,*psbN*,*psbT*,*psbZ*
	Rubisco	*rbcL*
Self-replication	DNA-dependent RNA polymerase	*rpoA*,*rpoB*,rpoC1*,*rpoC2*
	Large subunit of ribosome	rpl2*,*rpl14*,rpl16*,*rpl20*,*rpl22*,*rpl23*,*rpl32*,*rpl33*,*rpl36*
	Small subunit of ribosome	*rps2*,*rps3*,*rps4*,*rps7*,*rps8*,*rps11*,rps12*,*rps14*,*rps15*,rps16*,*rps18*,*rps19*
	Ribosomal RNAs	*rrn5*,*rrn4.5*,*rrn16*,*rrn23*
	Transfer RNAs	*trnA*-UGC*,*trnC*-*GCA*,*trnD*-*GUC*,*trnE*-*UUC*,*trnF*-*GAA*,*trnfM*-*CAU*,*trnG*-*GCC* *trnG*-UCC*,*trnH*-*GUG*,*trnI*-*CAU*,*trnI*-GAU*,*trnK*-UUU*,*trnL*-*CAA*,*trnL*-UAA*, *trnL*-*UAG*,*trnM*-*CAU*,*trnN*-*GUU*,*trnP*-*UGG*,*trnQ*-*UUG*,*trnR*-*ACG*,*trnR*-*UCU*, *trnS*-*GCU*,*trnS*-*GGA*,*trnS*-*UGA*,*trnT*-*GGU*,*trnT*-*UGU*,*trnV*-*GAC*,*trnV*-UAC*, trnW-CCA,trnY-GUA
Other genes	Maturase	*matK*
	Acetyl-CoA-carboxylase	*accD*
	C-type cytochrome synthesis	*ccsA*
	Envelope membrane protein	*cemA*
	ATP-dependent protease	clpP**
	Translational initiation factor	*infA*
Unknown functional genes	Conserved hypothetical open reading frames	*ycf1*,*ycf2*,ycf3**,*ycf4*,*ycf15*,*ycf68*

Intron-containing genes are marked by asterisks (*), *gene with one intron; **gene with two introns

### Repeat sequence analysis

**Fig. 2:**
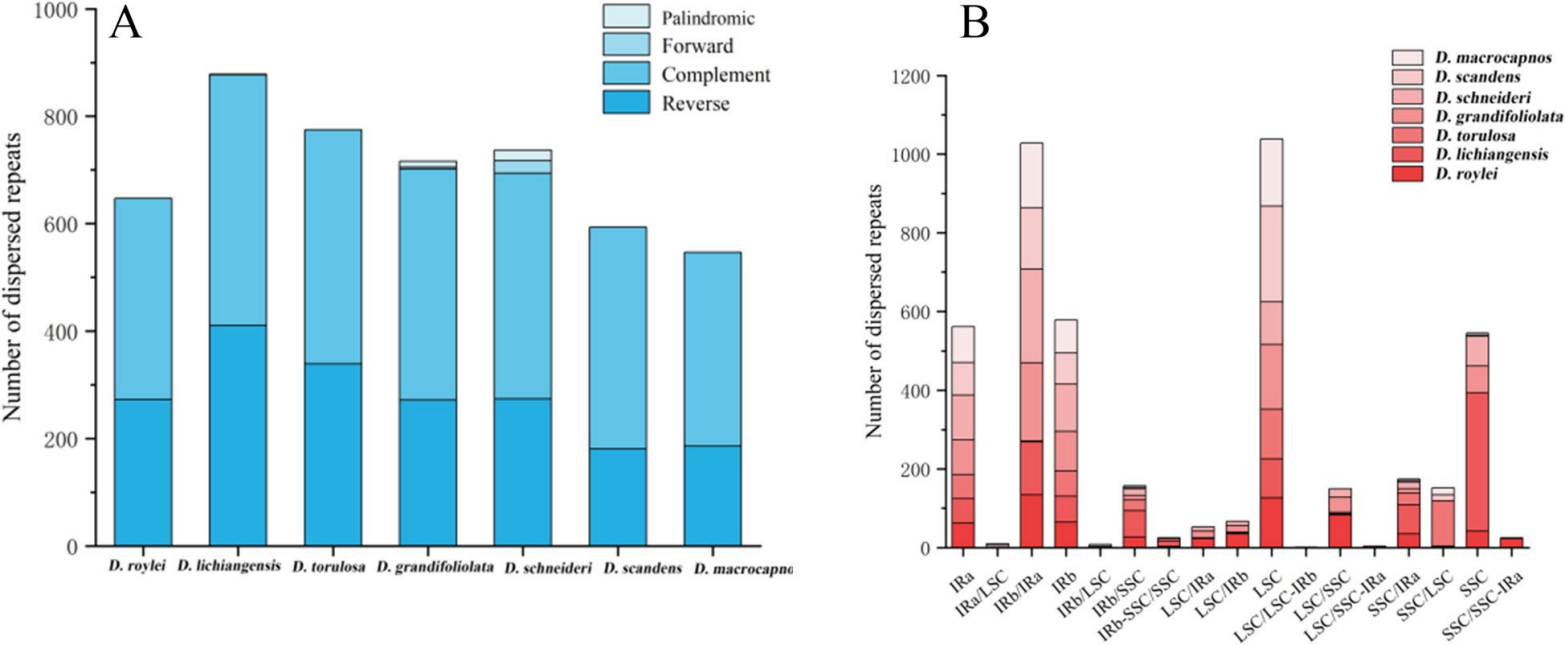
Dispersed repeated sequences analyses in cp genomes for seven *Dactylicapnos* species. **A** Statistics of four types of dispersed repeats sequences in seven cp genomes; **B **Distribution of dispersed repeats sequences in seven cp genomes

The studied cp genomes contained 546–878 dispersed repeats, including 360–467 forward repeats (F), 181–410 palindromic repeats (P), 3–23 complement repeats (C), and 1–20 reverse repeats (R), but some *Dactylicapnos* species do not have complement repeats and reverse repeats (Fig. 2A). Forward repeat was the most universal type, and most dispersed repeats were distributed in two-copy inverted repeat (IR) and large single-copy region (LSC) (Fig. 2B).

**Fig. 3:**
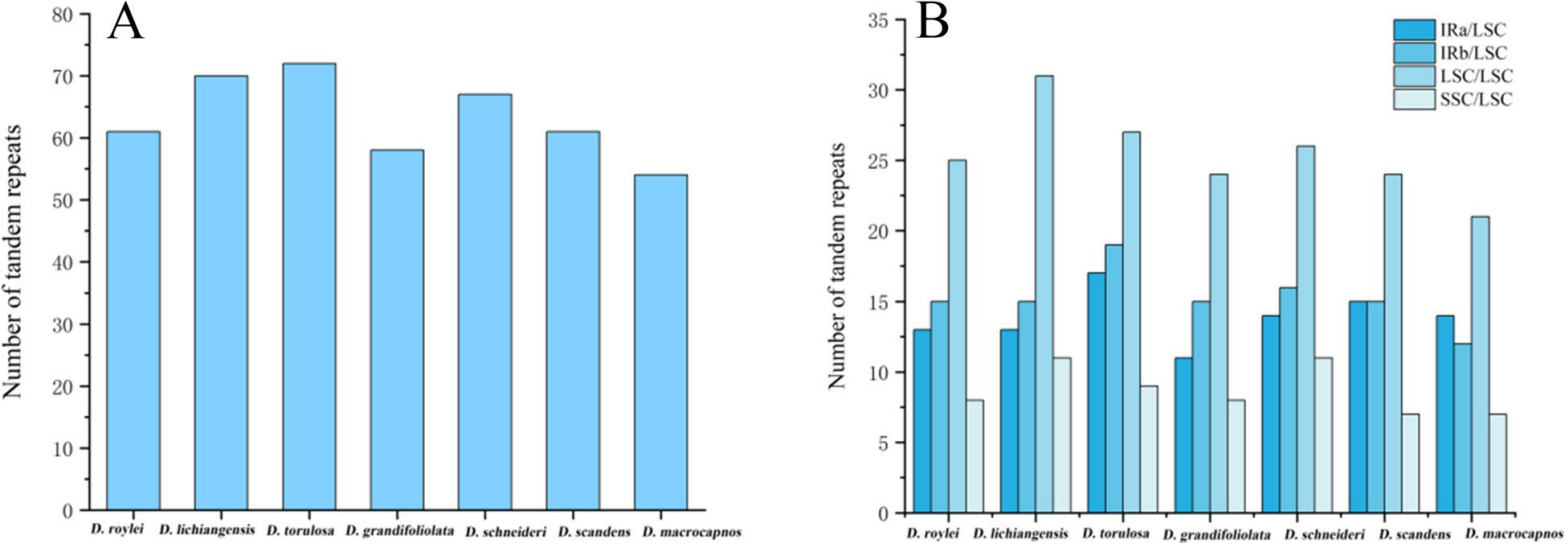
Tandem repeated sequence analyses for seven *Dactylicapnos* species. **A** Number of tandem repeats sequences in seven cp genomes; **B **Distribution of tandem repeats sequences in seven cp genomes

The number of the tandem repeats ranged in the studied cp genomes from 54 to 72. *Dactylicapnos torulosa* (Hook.f. & Thomson) Hutch. had the most tandem repeats and *Dactylicapnos macrocapnos* Hutch. had the smallest (Fig. 3A). There were 4 cases of distribution of tandem repeated in the region, distributed in IRa/LSC region, IRb/LSC region, LSC region, SSC/LSC region, most of the tandem repeats are distributed in LSC region (Fig. 3B).

### Simple sequence repeats (SSRs) analyses

**Fig. 4:**
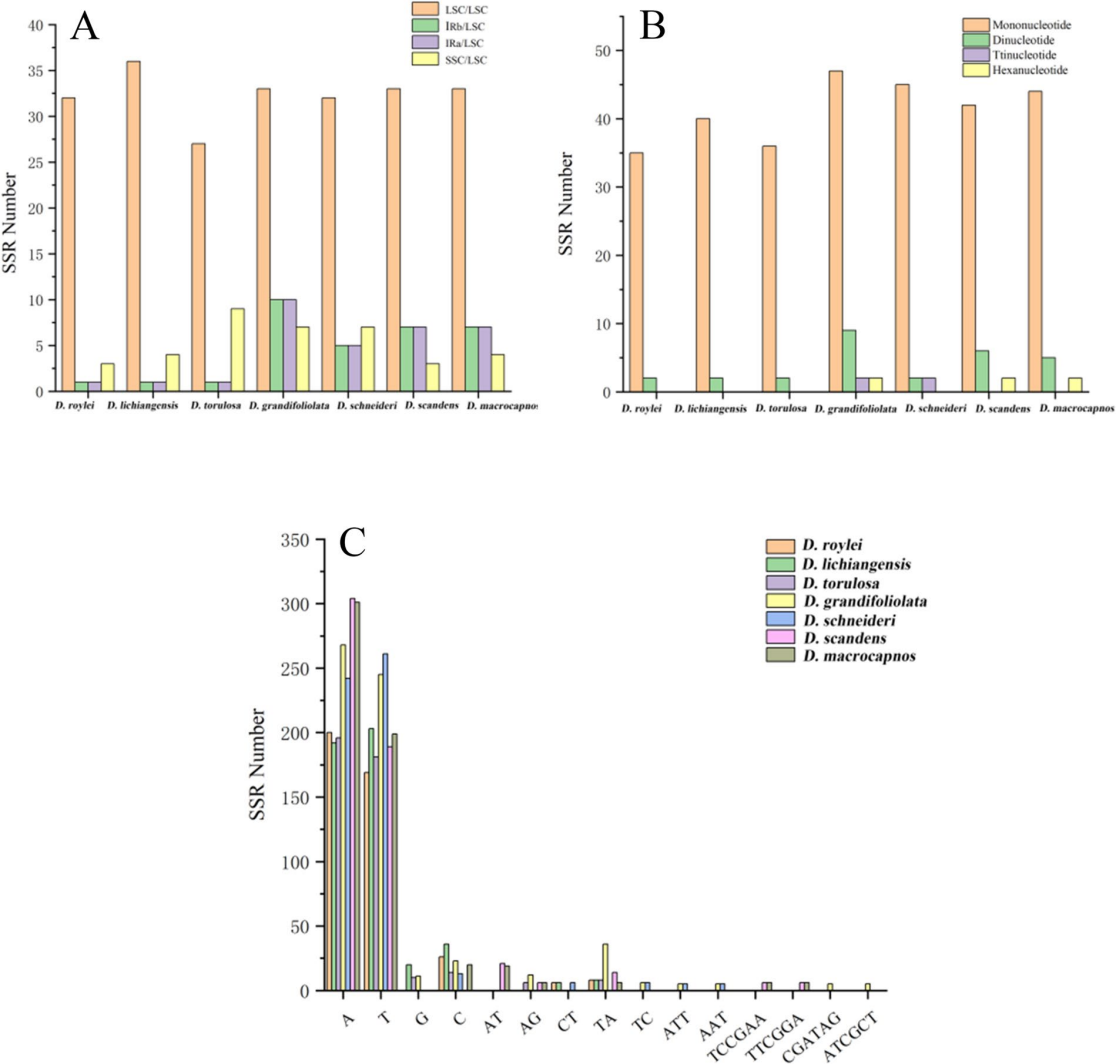
SSRs analyses for seven *Dactylicapnos* species. **A** The number of SSRs in LSC, SSC and IRs in seven cp genomes; **B **Number of different SSRs types; **C **Frequency of identified SSRs in different repeat class types

The SSRs were mainly distributed in the LSC region of *Dactylicapnos* species (Fig. 4A). A total of 327 SSRs were detected in the seven cp genomes, and the number of SSRs ranges from 37 (*Dactylicapnos roylei* Hutch.) to 60 (*D. grandifoliolata*), which had the largest number of mononucleotides (35–47), dinucleotides (2–9), trinucleotides (2), hexanucleotides (2), but some *Dactylicapnos* species did not have trinucleotides and hexanucleotides (Fig. 4B). These SSRs were dominated by mononucleotides (A/T) n. (Fig. 4C), suggesting that the base composition of SSRs is biased toward A/T base.

### Codon usage analysis

In total, 64 types of codons encoding 20 amino acids were detected, including three termination codons, UAA(*), UAG(*) and UGA(*). The number of codons ranged from 22,187 to 23,325, with the highest number of codons found in *D. schneideri*, and the lowest number of codons found in *Dactylicapnos lichiangensis* (Fedde) Hand.-Mazz..

**Fig. 5:**
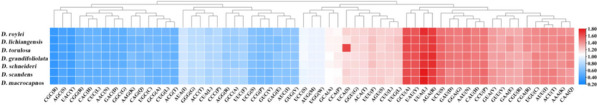
The RSCU analysis of 80 common CDS in the chloroplast genomes of *Dactylicapnos*

Relative synonymous codon usage (RSCU) values reflect a relationship between the number of actual codon emergence and the number of anticipated codon emergence [22], so that if the RSCU > 1, this mean that the condon has the strong preference. The RCUS calculated from 80 common CDS of the cp genomes of the studied species showed that all protein-coding sequence, 31 codons have RSCU > 1 (strong preference), 31 codons have RSCU < 1 (low preference), Methionine (Met) and threonine (Thr) have no bias (RSCU = 1). (Fig. 5).

### IR contraction and expansion

**Fig. 6:**
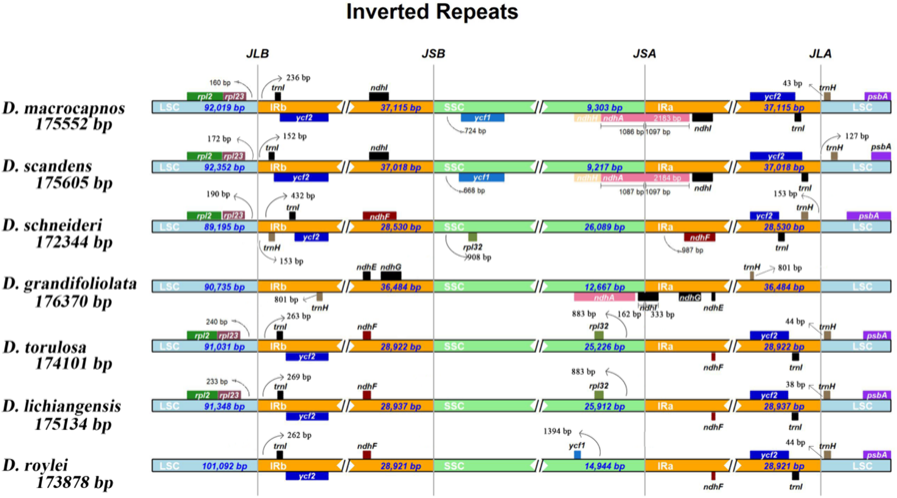
Comparison of LSC, IR, and SSC junction positions among seven *Dactylicapnos* species in cp genomes. JLB denotes the LSC/IRb junction,JSB denotes the SSC/IRb junction, JSA denotes the SSC/IRa junction, and JLA denotes the LSC/IRa junction

There were differences in the boundary regions of the studied species. In *D. macrocapnos, D. scandens, D. schneideri, D. torulosa,* and *D. lichiangensis*, *rpl23* was 160–240 bp to the left of the LSC/IRb boundary and *trnI* was 152–432 bp to the right of the LSC/IRb. The *ndhA* gene of *D. macrocapnos* and *D. scandens* covered the junction of SSC/IRa showed different sizes with 2183 bp and 2184 bp, extending into IRa by 1097 bp and SSC region by 1086 bp and 1087 bp. The *ndhI* gene of *D. grandifoliolata* also covered the junction of SSC/IRa, extending into IRa by 333 bp and SSC region by 162 bp. The gene *trnH* of *D. schneideri* and *D. grandifoliolata* was distributed on the left side of the border of IRa/LSC, and the gene *trnH* of the other species was distributed to the right of the IRa/LSC junction, with an interval of 38–127 bp from the border to the gene (Fig. 6).

### Similarity analysis and synteny analysis

**Fig. 7:**
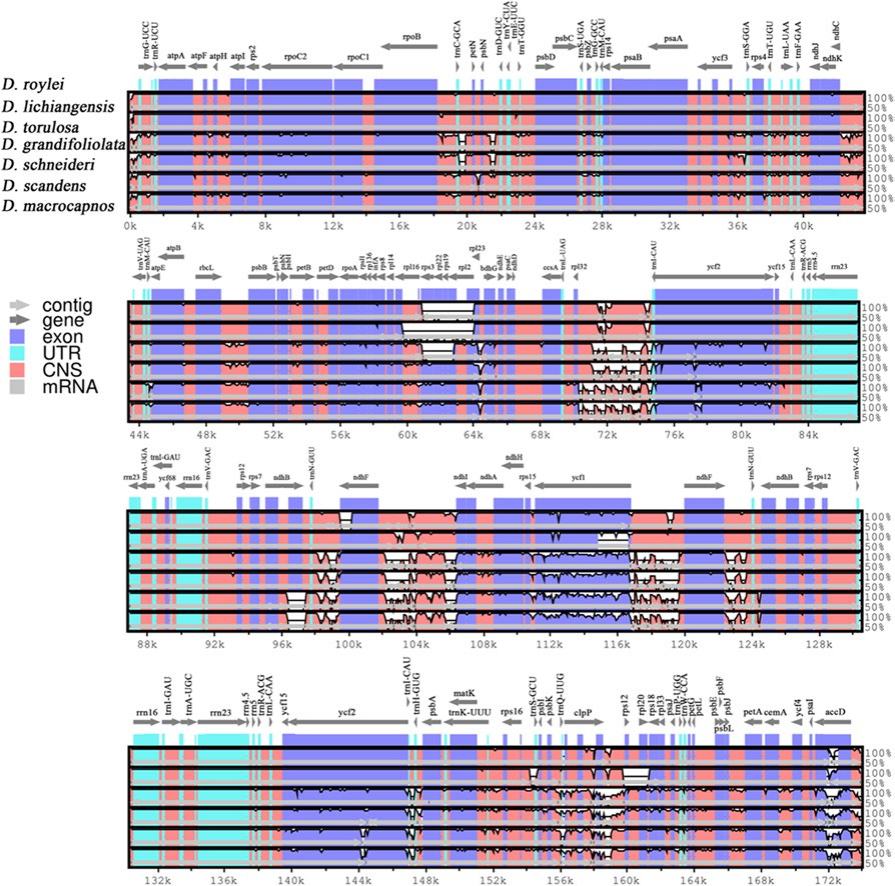
Visualization of genome alignment of the chloroplast genomes of seven *Dactylicapnos* species using *D. roylei* as a reference by mVISTA. The x-axis represents the coordinate in the chloroplast genome. The Y-axis represents different species, and sequence similarity of aligned regions is displayed as horizontal bars, which expresses as a percentage within 50–100%

Analysis of the level of divergence among the studied species sequences, with *D. roylei* as a reference, done by mVISTA revealed that the bulk of among sequence variation is located in non-coding intergenic regions and that there were apparent deletions between the coding genes *rps3*–*rpl2* of *D. lichiangensis, D. torulosa* and *D. grandifoliolata* (Fig. 7).

**Fig. 8:**
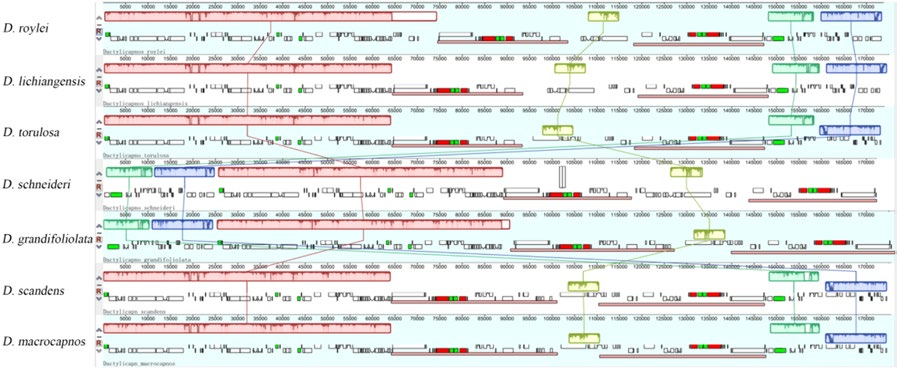
Synteny analysis for seven *Dactylicapnos* species in chloroplast genomes

The synteny analysis revealed some genomic rearrangements and inversions in the seven cp genomes. Due to the expansion of IR region, the nucleotide sequences of in cp genomes of *D. schneideri* and *D. grandifiliolata* was rearranged, and some single copy regions of *D. torulosa*, *D. macrocapnos*, *D. scandens* and *D. grandifoliolata* were inverted (Fig. 8).

### Phylogenetic analysis

**Fig. 9:**
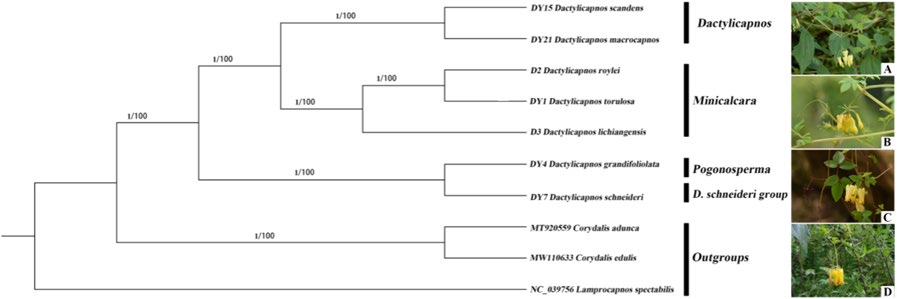
Maximum likelihood and Bayesian inference phylogeny of *Dactylicapnos* based on 78 common CDSs of 10 chloroplast genomes. From left to right, Numbers above branches indicate Bayesian posterior probability [BIPP], and Maximum Likelihood bootstrap support [MLBS], respectively. (**A**, *D. macrocapnos*; **B**, *D. torulosa*; **C**, *D. grandifoliolata*; **D**, *D. schneideri*)

The maximum likelihood (ML) and bayesian inference (BI) phylogenetic trees (Fig. 9) were constructed using 78 common CDS of the cp genomes of 10 Fumarioideae species, including seven newly sequenced *Dactylicapnos* species and three outgroups including *Lamprocapnos spectabilis* (L.) Fukuhara, *Corydalis adunca* Maxim. and *Corydalis edulis* Maxim.. The ML and BI methods yielded identical tree topologies with full support for each node (MLBS = 100% and BIPP = 1). The genus was found to be monophyletic and the species of *Dactylicapnos* formed three distinct clades. The clade consistsing of *D. schneideri*, and *D. grandifoliolata* were sister to the rest of *Dactylicapnos*. *D. schneideri* formed an independent informal group, and was clustered together with *D. grandifoliolata* of sect. *Pogonosperma*. Section *Dactylicapnos* including *D. scandens* and *D. macrocapnos* were sister to sect. *Minicalcara* including *D. lichiangensis*, *D. roylei* and *D. torulosa* with full support (MLBS = 100% and BIPP = 1).

## Discussion

### Structure and comparative analysis of *Dactylicapnos* species

Comparative analysis of cp genomes has been widely used in many plant taxa [23]. In this study, the cp genome of seven *Dactylicapnos* species were first sequenced, it is also the first time to explore *Dactylicapnos* species from the molecular analysis. As in the most angiosperms, the cp genome of *Dactylicapnos* has a typical quadripartite structure [14] but is very long 172,322–176,370 bp being one of the largest cp genomes sequenced to date [24], and the genomic size of the SSC region ranges from 9303 bp to 26,089 bp, with a number difference of about 16 kb, indicating the weakest conservatism and stability. The seven cp genomes are similar in structure, and had from 133 to 140 genes, which indicates that *Dactylicapnos* cp genomes are structurally conserved and rich in genetic information, which is a reliable molecular material for phylogenetic studies.

The highly conservative IR region is thought to play an important role in stabilizing the chloroplast genome structure [25]. Expansion and contraction of the IR region is a common phenomenon in plant evolutionary history responsible for cp genome length variation [26], which affects the cp genome’s rate of evolution [27, 28], examples are early-diverging eudicots [29, 30] and Apiales [31]. There have been many research about the expansion and contraction of the IR region, and the expansion mechanism of the IR region, the major viewpoint is that minor and apparently random IR expansion may be caused by gene conversion, and larger IR expansion may be achieved through double-strand DNA breaks and subsequent repair mechanism [32, 33], and the contraction mechanism of the IR region is also assumed to be the double-strand DNA breaks and subsequent repair mechanism [34]. In the present study, the IR region has significant expansion or contraction, forming a variety of boundary genes, and the seven cp genomes can be divided into three types according to their variability, which are consistent with the clustering results of the phylogenetic analysis. The gene location information in the boundary region can reveal the phylogenetic relationships between species to some extent [35]. In addition, as the expansion of the IR region at the LSC-IRb boundary, the *trnH* gene of *D. schneideri* and *D. grandifoliolata* entered the IR region leading to genomic rearrangement of these two sequences and the *trnH* gene becames gene with two copies. The chloroplast genome has multiple copies in the cell and has sufficient interspecific differentiation [35], chloroplast genome sequences for species identification is one of the best methods at present [36], while the cp genome of *Dactylicapnos* species have significant differences in expansion and contraction, and there are obvious differences in the size of the LSC, SSC, and IR regions of seven cp genomes, suggesting that *Dactylicapnos* species have a high degree of interspecies differentiation, which can be utilized to adequately demonstrate the phylogenetic relationships between *Dactylicapnos* species through the cp genomes.

### Repeat sequences and SSRs

The plastid genome contains many oligonucleotide repeat sequences that are considered biomarkers of mutational hotspots [37, 38]. Repeat sequences have an important position in genome rearrangements and an important molecular marker in phylogenetic studies [39, 40]. In this present study, four different types of repeat sequences were detected, with the highest number of forward repeats (F) and the lowest number of complement repeats (C). The composition of different types of repeat sequences affects the inheritance and evolution of species [41]. There are small differences in the number and type of repeats among closely related species, both *D. schneideri* and *D. grandifoliolata* have four types of repetitive sequences with high similarity in type and number, inferring that the two species may have similarities in genetics and evolution [42]. SSRs are repeated DNA motifs with 1–6 nucleotides and have high polymorphism rates at the species level, have been extensively investigated in population genetics, phylogeography and variety identification [43, 44]. In this study, we found that the types of SSRs in seven cp genomes were found to be essentially the same, but the number of sequences contained in each type was different. Most SSRs loci were distributed in LSC region, with size ranging from 10–125 bp. The mononucleotide (A/T) was the highest proportion in the cp genomes of seven *Dactylicapnos* species, were found in all species. SSRs polymorphisms are repeat length polymorphisms caused by elongation or shortening of repeat units [45], it is a common molecular tool used to study the evolution of species. In the *Camellia* [46] and *Triticum* [47] plant, genetic diversity analysis was performed by amplifying SSRs primers, which led to the construction of genetic evolutionary relationships among species. The large number of SSRs detected in this research can be used as potential molecular markers for subsequent studies of *Dactylicapnos* species and also provide a theoretical basis for interspecific identification.

### Codon usage analysis

The codons that encode the same amino acid are called synonymous codons [48]. In the process of species evolution, synonymous codons are not only associated with nature selection, mutation and genetic drift [49, 50], but also affected by factors such as genome size [51], tRNA abundance [52, 53] and gene expression levels [54], resulting in the genetic codes of different species tend to use one of several synonymous codons, called codon usage bias, which a common feature of eukaryotic genomes and is essential for the regulation of gene expression [55]. The results of the codon usage analysis showed that 31 codons had RSCU values > 1, indicating a codon bias in the amino acids, but unlike other dicotyledons plants [56], these 31 codons of *Dactylicapnos* do not prefer to end in A/U, It is possible that different levels of evolutionary pressures in *Dactylicapnos* species have biased the use of codons in this chloroplast genome, but the mechanisms involved need to be further explored [57, 58].

### Phylogenetic analysis

The cp genome sequences have been successfully used to reveal phylogenetic relationships [59]. However, due to the different degree of gene rearrangement and inversion in *Dactylicapnos*, there are significant differences in gene order between sequences, and reliable phylogenetic relationships could not be established using the whole chloroplast genome. The analysis of 78 common CDS from the cp genomes of seven *Dactylicapnos* species and three outgroups showed that seven *Dactylicapnos* species were divided into three major clades with full support. Recent classification of *Dactylicapnos* based on morphology divided the genus into three sections and two informal groups [5]. Our study covered all three sections and one informal group, and our results basically clarified the infrageneric relationships between these three sections and one informal group. The first separated clade includes *D. schneideri* of an independent informal group sensu Lidén and Pathak [5] and *D. grandifoliolata* of sect. *Pogonosperma* sensu Lidén and Pathak [5]. The cp genomes of both *D. schneideri* and *D. grandifoliolata* had genomic rearrangements and contracted in the IR regions, and were clustered into the same clade, indicationg their close genetic relationship. The other two clades correspond to the two sections sensu Lidén and Pathak [5], sect. *Dactylicapnos* and sect. *Pogonosperma*, respectively. There were differences in the number of genes and GC content of these two clades, and there were also obvious differences in morphological characteristics. *D. macrocapnos* and *D. scandens* which were perennial plants with cylindrical stems and small flat globular elaiosomes [2], while *D. torulosa*, *D. roylei* and *D. lichiangensis* were all annual plants with winged-ridged stems and irregular mass elaiosomes [3]. *D. scandens* and *D. macrocapnos* of sect. *Dactylicapnos* were clustered together with full support, and *D. lichiangensis*, *D. roylei* and *D. torulosa* of sect. *Minicalcara* were also clustered together, so we confirmed sect. *Dactylicapnos* and sect. *Minicalcara* based on the plastome phylogenomics.

## Conclusion

The cp genome of *Dactylicapnos* species had a typical tetrad structure and high sequence conservation. A total of 133–140 genes were annotated in the seven *Dactylicapnos* species, and a large number of repeat sequences and SSRs detected were important molecular markers in population genetics and phylogenetics. Expansion of IR regions and genomic rearrangements revealed by comparative genomic analysis played an important role in the evolution of *Dactylicapnos* species, and showed that the cp genomes of the *D. macrocapnos* and *D. scandens* were closer in structural variation, the *D. schneideri* was similar to and *D. grandifoliolata*, while the *D. torulosa*, *D. lichiangensis* and *D. roylei* were more consistent, which supported the results of phylogenetic analyses that categorized the seven species of *Dactylicapnos* into three clades. In addition, the most comprehensive and robust phylogeny covering all three sections and one informal group of *Dactylicapnos* based on cp genomes was reconstructed to basically clarify infrageneric relationships for the first time. Phylogenetic analysis showed that seven species separated into three major evolutionary clades, which suggested that this genus should be divided into three sections. The novel genomic resources provided here will aid future study in development of medicine resources, infrageneric classification, character evolution, diversification and biogeography. It also showed that the structural information and variation of chloroplast genomes were important for phylogenetic analysis, providing strong evidence for a deeper understanding of phylogenetic relationships and evolution among species.

## Materials and methods

### Plant material, DNA extraction and sequencing

Most of the material of *Dactylicapnos* species were fresh leaves collected in the field and dried with silica gel, and a few materials were obtained from the herbarium of KUN (Herbarium, Kunming Institute of Botany, CAS) and PE (Herbarium, Institute of Botany,CAS) (Table 3). The DNA extraction, library preparation and shallow sequencing were performed by Novogene, and the library was sequenced on the Illumina Hiseq 4000 platform with 150 bp paired-end reads. For the herbarium specimens, the method of Zeng et al. [60] was adopted for sequencing and library construction.

**Table 3:** Species Collection Information

Species	Herbarium	Collection number	Determinavit	Locality	coordinate information (Lat, Lon)	GenBank accession
*D*. *macrocapnos*	Herbarium, Institute of Botany, CAS (PE)	01029	Juntong Chen, Shunquan Yang	Nyalam, Xizang, China	27.97°, 85.96°	OR589107
*D*. *scandens*	Herbarium, Kunming Institute of Botany, CAS (KUN)	LuJL118	Juntong Chen, Shunquan Yang	PingBian, Wenshan Zhuang and Miao Autonomous Prefecture, Yunnan, China	23.13°, 104.78°	OR568573
*D*. *schneideri*	Herbarium, Kunming Institute of Botany, CAS (KUN)	10,566	Juntong Chen, Shunquan Yang	Yanyuan, Liangshan Yi Autonomous Prefecture, Sichuan, China	27.56°,101.75°	OR589106
*D*. *grandifoliolata*	Herbarium, Kunming Institute of Botany, CAS (KUN)	Deng-15218	Juntong Chen, Shunquan Yang	Yadong, Rikaze, Xizang, China	27.24°, 89.02°	OR589105
*D*. *torulosa*	Herbarium, Kunming Institute of Botany, CAS (KUN)	SunH-07ZX-3200	Juntong Chen, Shunquan Yang	Yulong, Lijiang, Yunnan, China	26.79°, 99.64°	OR589104
*D*. *lichiangensis*	Herbarium, Kunming Institute of Botany, CAS (KUN)	SunH-07ZX-3234	Juntong Chen, Shunquan Yang	Yulong, Lijiang, Yunnan, China	26.78°, 99.67°	OR589103
*D*. *roylei*	Herbarium, Kunming Institute of Botany, CAS (KUN)	38,434	Juntong Chen, Shunquan Yang	Xiaojin, Sichuan, China	30.99°, 102.69°	OR568572

### Chloroplast genome assembly, annotation and codon usage

De novo assembly of the cp genome was carried out using GetOrganelle 1.7.6.1 [61]. We used the genome annotator PGA [62] to annotate the sequences that have been assembled into loops using the *Lamprocapnos spectabilis* (NC_039756) as the reference, and manually correct the position of the start and stop codons and the boundary between the exons and introns with Geneious Prime 2023.0.4 [63]. Finally, the physical maps of cp genome were created by using OrganellarGenomeDRAW (https://chlorobox.mpimp-golm.mpg.de/OGDraw.html) [64]. The RSCU was the ratio of the frequency of a specific codon to the expected frequency of that codon, which was obtained by Genepioneer platform, and plotted the heatmap of RSCU values with TBtools 1.116 [65].

### Analysis of repeat sequences and SSRs

Repeat sequences in the cp genome were detected by REPuter [66], including forward, palindromic, reverse and complement repeats, the parameters were set with minimum repeat size 30 bp, and an hamming distance of 3. And exploring tandem repeats of cp genome by the Tandem Repeat Finder [67]. The simple sequence repeats (SSRs) were identified by using MISA online tool (https://webblast.ipk-gatersleben.de/misa/) [68], and the repeat thresholds for mononucleotide, dinucleotide, trinucleotide, tetrtanucleotide, pentanucleotide and hexanucleotide SSRs were 10, 6, 5, 5, 5, 5, respectively.

### Comparative genomic analyses

The online program IRscope (https://irscope.shinyapps.io/irapp/) [69] was used to study the expansion and contraction of the IR region in the cp genome sequence of *Dactylicapnos* species. The genome comparison of the seven *Dactylicapnos* species in the cp genomes was analyzed by the mVISTA (https://genome.lbl.gov/vista/index.shtml) [70] program with the Shuffle-LAGAN mode, and the synteny analysis of cp genome was performed with Mauve [71].

### Phylogenetic analysis

Phylogenetic analysis was performed based on 78 common CDS of the cp genomes of 10 Fumarioideae species, including seven *Dactylicapnos* cp genomes and three closely related species (*Lamprocapnos spectabilis*, *Corydalis adunca* and *Corydalis edulis*). These three species were selected as outgroups based on previous phylogenetic results[10–12], and these three plastomes were downloaded from GenBank. All sequences were aligned using MAFFT and maximum likelihood (ML) analysis was performed by RAxML-8.2.12 on CIPRES (https://www.phylo.org/portal2/) website with the GTRGAMM model, and 1000 bootstrap replicates. The best-fit model GTR + I + G was selected by AIC (Akaike Information Criterion) with jModelTest 2.1.10 [72], and the Bayesian inference (BI) analyses were conducted by MrBayes-3.2.7 on CIPRES website, with the settings: four MCMC simulations were run simultaneously and sampled every 1,000 generations for a total of two million generations, the first 25% of trees were discarded as burn-in.

## Data Availability

The datasets generated and analysed during the current study are available in the National Center for Biotechnology Information (NCBI) database, using the accession number OR568572, OR568573, OR589103, OR589104, OR589105, OR589106 and OR589107 (see Table 3 for details).

## References

[1] Wallich N. Tentamen florae Napalensis illusrata, consisting of botanical descriptions and lithographic figures of select Nipal plants. Asiatic Lithographic Press. 1826;2:51–2.

[2] Zhang ML, Su ZY, Lidén M. Papaveraceae. In: Wu ZY, Raven PH, Hong DY, editors. Flora of China. Vol 7. Beijing: Science Press; 2008. p. 291–295.

[3] Wu ZY, Zhang X, Su ZY (1999). Flora Reipublicae Popularis Sinicae. Beijing Science Press..

[4] Lidén M. Three new species of *Dactylicapnos* (Fumariaceae) and a synopsis of the *D. macrocapnos* complex. Nord J Bot. 2010;28(6):656–60.

[5] Lidén M, Pathak MK. Studies in *Dactylicapnos* (Papaveraceae–Fumarioideae) part II Revision of *Dactylicapnos* sect. *Pogonosperma* sect. nov with *D.**arunachalensis* sp nov. Nord J Bot. 2014;32(2):176–84.

[6] Wang FH, Hu X, Chen HL, Ma JP, Wang JX, Hou AJ (2009). Alkaloids from *Dactylicapnos scandens* Hutch. China J Chin Materia Med.

[7] Guo CC. The metabolism and pharmacokinetics of isocorydine and protopine in *Dactylicapnos scandens*. Zhejiang University; 2013. p. 2–7.

[8] Wang B, Zhao YJ, Zhao YL, Liu YP, Li XN, Zhang HB, Luo XD (2019). Exploring aporphine as anti-inflammatory and analgesic lead from *Dactylicapnos scandens*. Org Lett.

[9] Lidén M, Fukuhara T, Rylander J, Oxelman B (1997). Phylogeny and classification of Fumariaceae, with emphasis on *Dicentra* sl, based on the plastid gene *rps16* intron. Plant Syst Evol.

[10] Perez-Gutierrez MA, Romero-Garcia AT, Salinas MJ, Blanca G, Fernandez MC, Suarez-Santiago VN (2012). Phylogeny of the tribe Fumarieae (Papaveraceae s.l.) based on chloroplast and nuclear DNA sequences: evolutionary and biogeographic implications. American Journal Botany..

[11] Perez-Gutierrez MA, Romero-Garcia AT, Fernandez MC, Blanca G, Salinas-Bonillo MJ, Suarez-Santiago VN (2015). Evolutionary history of fumitories (subfamily Fumarioideae, Papaveraceae): An old story shaped by the main geological and climatic events in the Northern Hemisphere. Molecular Phylogenetic and Evolution.

[12] Chen JT, Lidén M, Huang XH, Zhang L, Zhang XJ, Kuang TH, Landis JB, Wang D, Deng T, Sun H. An updated classification for the hyper-diverse genus *Corydalis* (Papaveraceae: Fumarioideae) based on phylogenomic and morphological evidence. J Integr Plant Biol. 2023;65(9):2138–56. 10.1111/jipb.13499 37119474

[13] Palmer JD (1985). Comparative organization of chloroplast genomes. Annu Rev Genet.

[14] Chumley TW, Palmer JD, Mower JP, Fourcade HM, Calie PJ, Boore JL, Jansen RK (2006). The complete chloroplast genome sequence of *Pelargonium*× *hortorum*: organization and evolution of the largest and most highly rearranged chloroplast genome of land plants. Mol Biol Evol.

[15] Shinozaki K, Ohme M, Tanaka M, Wakasugi T, Hayashida N, Matsubayashi T, Zaita N, Chunwongse J, Obokata J, Yamaguchi-Shinozaki K, Ohto C, Torazawa K, Meng BY, Sugita M, Deno H, Kamogashira T, Yamada K, Kusuda J, Takaiwa F, Kato A, Tohdoh N, Shimada H, Sugiura M (1986). The complete nucleotide sequence of the tobacco chloroplast genome: its gene organization and expression. EMBO J.

[16] Hiratsuka J, Shimada H, Whittier R, Ishibashi K, Sakamoto M, Mori M, Kondo C, Honji Y, Sun CR, Meng BY, Li YQ, Kanno A, Nishizawa Y, Hirai A, Shinozaki K, Sugiura M. The complete sequence of the rice (*Oryza sativa*) chloroplast genome: intermolecular recombination between distinct tRNA genes accounts for a major plastid DNA inversion during the evolution of the cereals. Mol Gen Genet MGG. 1989;217:185–94. 10.1007/BF02464880 2770692

[17] Ohyama K, Fukuzawa H, Kohchi T, Shirai H, Sano T, Sano S, Umesono K, Shiki Y, Takeuch M, Chang Z, Aota SI, Inokuch H, Ozek H (1986). Chloroplast gene organization deduced from complete sequence of liverwort Marchantia polymorpha chloroplast DNA. Nature.

[18] Dong WP, Xu C, Cheng T, Lin K, Zhou S (2013). Sequencing angiosperm plastid genomes made easy: a complete set of universal primers and a case study on the phylogeny of Saxifragales. Genome Biol Evol.

[19] Clegg MT, Gaut BS, Learn GH, Morton BR (1994). Rates and patterns of chloroplast DNA evolution. Proc Natl Acad Sci.

[20] Yang Z, Wang GX, Ma Q, Ma WX, Liang LS, Zhao TT (2019). The complete chloroplast genomes of three Betulaceae species: implications for molecular phylogeny and historical biogeography. PeerJ.

[21] Wang YH, Wang S, Liu YL, Yuan QL, Sun JH, Guo LP (2021). Chloroplast genome variation and phylogenetic relationships of *Atractylodes* species. BMC genomics..

[22] Zhou JH, Zhang J, Chen HT, Ma LN, Liu YS (2010). Analysis of synonymous codon usage in foot-and-mouth disease virus. Vet Res Commun.

[23] Fan XG, Wang WC, Wagutu GK, Li W, Li XL, Chen YY (2022). Fifteen complete chloroplast genomes of *Trapa* species (Trapaceae): insight into genome structure, comparative analysis and phylogenetic relationships. BMC Plant Biol.

[24] Hong Z, Wu ZQ, Zhao KK, Yang ZJ, Zhang NN, Guo JY, Tembrock LR, Xu DP (2020). Comparative analyses of five complete chloroplast genomes from the genus *Pterocarpus* (Fabacaeae). Int J Mol Sci.

[25] Maréchal A, Brisson N (2010). Recombination and the maintenance of plant organelle genome stability. New Phytologist Foundation.

[26] Wang RJ, Cheng CL, Chang CC, Wu TM, Chaw SM (2008). Dynamics and evolution of the inverted repeat-large single copy junctions in the chloroplast genomes of monocots. BMC Evol Biol.

[27] Kim KJ, Lee HL (2004). Complete chloroplast genome sequences from Korean ginseng (*Panax schinseng* Nees) and comparative analysis of sequence evolution among 17 vascular plants. DNA Res.

[28] Zhang HY, Li C, Miao HM, Xiong SJ (2013). Insights from the complete chloroplast genome into the evolution of *Sesamum indicum* L. PLoS ONE.

[29] Sun Y, Moore MJ, Zhang S, Soltis PS, Soltis DE, Zhao T, Meng A, Li X, Li J, Wang H (2016). Phylogenomic and structural analyses of 18 complete plastomes across nearly all families of early-diverging eudicots, including an angiosperm-wide analysis of IR gene content evolution. Mol Phylogenet Evol.

[30] Sun YX, Moore MJ, Meng AP, Soltis PS, Soltis DE, Li JQ, Wang HC (2013). Complete plastid genome sequencing of Trochodendraceae reveals a significant expansion of the inverted repeat and suggests a Paleogene divergence between the two extant species. PLoS ONE.

[31] Downie SR, Jansen RK (2015). A comparative analysis of whole plastid genomes from the Apiales: expansion and contraction of the inverted repeat, mitochondrial to plastid transfer of DNA, and identification of highly divergent noncoding regions. Syst Bot.

[32] Goulding SE, Wolfe KH, Olmstead RG, Morden CW (1996). Ebb and flow of the chloroplast inverted repeat. Mol Gen Genet MGG.

[33] Wang RJ, Cheng CL, Chang CC, Wu CL, Su TM, Chaw SM (2008). Dynamics and evolution of the inverted repeat-large single copy junctions in the chloroplast genomes of monocots. BMC Evol Biol.

[34] Peery RM. Understanding angiosperm genome interactions and evolution: insights from sacred lotus (Nelumbo nucifera) and the carrot family (Apiaceae). University of Illinois at Urbana-Champaign; 2015. p. 11–54.

[35] Chen MM, Zhang M, Liang ZS, He QL (2022). Characterization and Comparative Analysis of Chloroplast Genomes in Five *Uncaria* Species Endemic to China. Int J Mol Sci.

[36] Hollingsworth PM, Graham SW, Little DP (2011). Choosing and using a plant DNA barcode. PLoS ONE.

[37] Ibrar A, Biggs PJ, Matthews PJ, Collins LJ, Hendy MD, Lockhart PJ (2012). Mutational Dynamics of Aroid Chloroplast Genomes. Genome Biol Evol.

[38] Jungeun L, Kang Y, Chul SS, Hyun P, Hyoungseok L (2014). Combined Analysis of the Chloroplast Genome and Transcriptome of the Antarctic Vascular Plant *Deschampsia antarctica* Desv. PLoS ONE.

[39] Cavalier-Smith T (2002). Chloroplast evolution: secondary symbiogenesis and multiple losses. Curr Biol.

[40] Nazareno AG, Carlsen M, Lohmann LG (2015). Complete chloroplast genome of *Tanaecium tetragonolobum*: the first Bignoniaceae plastome. PLoS ONE.

[41] Keller J, Rousseau-Gueutin M, Martin GE, Morice J, Boutte J, Coissac E, Ourari M, Aïnouche M, Salmon A, Cabello-Hurtado F, Aïnouche A (2017). The evolutionary fate of the chloroplast and nuclear *rps16* genes as revealed through the sequencing and comparative analyses of four novel legume chloroplast genomes from *Lupinus*. DNA Res.

[42] He Y, Xiao HT, Deng C, Xiong L, Yang J, Peng C (2016). The complete chloroplast genome sequences of the medicinal plant *Pogostemon cablin*. Int J Mol Sci.

[43] Xue JH, Wang S, Zhou SL (2012). Polymorphic chloroplast microsatellite loci in *Nelumbo* (Nelumbonaceae). Am J Bot.

[44] Zheng G, Wei LL, Ma L, Wu ZQ, Gu CH, Chen K (2020). Comparative analyses of chloroplast genomes from 13 *Lagerstroemia* (Lythraceae) species: identification of highly divergent regions and inference of phylogenetic relationships. Plant Mol Biol.

[45] Sonah H, Deshmukh RK, Sharma A, Singh VP, Gupta DK, Gacche RN, Rana JC, Singh NK, Sharma TR (2011). Genome-wide distribution and organization of microsatellites in Plants: An insight into marker development in *Brachypodium*. PLoS ONE.

[46] Wang LY, Liu BY, Jiang YH, Duan YS, Cheng H, Zhou J, Tang YC (2009). Phylogenetic analysis of interspecies in section *Thea* through SSR markers. J Tea Sci.

[47] Shi JX, Qiao YL, Ma YQ, Ji WQ, He PR, Weng YJ. Analysis on genetic evolution relation of A, B genomes between *Triticum**aestivum* and *T*. *dicoccoides* by SSR. Acta Botanica Boreali Occidentalia Sinica. 2003;23(06):933–7.

[48] Lagerkvist ULF (1978). " Two out of three": an alternative method for codon reading. Proc Natl Acad Sci.

[49] Bulmer M (1991). The selection-mutation-drift theory of synonymous codon usage. Genetics.

[50] Hershberg R, Petrov DA (2008). Selection on codon bias. Annu Rev Genet.

[51] Dong LN, Du XY, Zhou W (2019). The complete plastid genome sequence of *Begonia guangxiensis*. Mitochondrial DNA Part B.

[52] Duret L. tRNA gene number and codon usage in the *C*. *elegans* genome are co-adapted for optimal translation of highly expressed genes. Trends in Genetics. 2000;16(7):287–9. 10.1016/s0168-9525(00)02041-2 10858656

[53] Olejniczak M, Uhlenbeck OC (2006). tRNA residues that have coevolved with their anticodon to ensure uniform and accurate codon recognition. Biochimie.

[54] Hiraoka Y, Kawamata K, Haraguchi T, Chikashige Y (2010). Codon usage bias is correlated with gene expression levels in the fission yeast *Schizosaccharomyces pombe*. Genes Cells.

[55] Lyu XL, Liu Y (2020). Nonoptimal codon usage is critical for protein structure and function of the master general amino acid control regulator CPC-1. Molecular Biology and Physiology.

[56] Kawabe A, Miyashita NT (2003). Patterns of codon usage bias in three dicot and four monocot plant species. Genes Genet Syst.

[57] Kong WQ, Yang JH (2017). The complete chloroplast genome sequence of *Morus cathayana* and *Morus multicaulis*, and comparative analysis within genus *Morus L*. PeerJ.

[58] Suzuki H, Morton BR (2016). Codon adaptation of plastid genes. PLoS ONE.

[59] Moore MJ, Bell CD, Soltis PS, Soltis DE (2007). Using plastid genome-scale data to resolve enigmatic relationships among basal angiosperms. Proc Natl Acad Sci.

[60] Zeng CX, Hollingsworth PM, Yang J, He ZS, Zhang ZR, Li DZ, Yang JB (2018). Genome skimming herbarium specimens for DNA barcoding and phylogenomics. Plant Methods.

[61] Jin JJ, Yu WB, Yang JB, Song Y, de Pamphilis CW, Yi TS, Li DZ (2020). GetOrganelle: a fast and versatile toolkit for accurate de novo assembly of organelle genomes. Genome Biology..

[62] Qu XJ, Moore MJ, Li DZ, Yi TS (2019). PGA: a software package for rapid, accurate, and flexible batch annotation of plastomes. Plant Methods.

[63] Kearse M, Moir R, Wilson A, Stones-Havas S, Cheung M, Sturrock S, Buxton S, Cooper A, Markowitz S, Duran C, Thierer T, Ashton B, Meintjes P, Drummond A (2012). Geneious Basic: an integrated and extendable desktop software platform for the organization and analysis of sequence data. Bioinformatics.

[64] Lohse M, Drechsel O, Bock R (2007). OrganellarGenomeDRAW (OGDRAW): a tool for the easy generation of high-quality custom graphical maps of plastid and mitochondrial genomes. Curr Genet.

[65] Chen C, Chen H, Zhang Y, Thomas HR, Frank MH, He YH, Xia R (2020). TBtools: an integrative toolkit developed for interactive analyses of big biological data. Molecular Plant..

[66] Kurtz S, Schleiermacher C (1999). REPuter: fast computation of maximal repeats in complete genomes. Bioinformatics (Oxford, England).

[67] Benson G (1999). Tandem repeats finder: a program to analyze DNA sequences. Nucleic Acids Res.

[68] Beier S, Thiel T, Münch T, Scholz U, Mascher M (2017). MISA-web: a web server for microsatellite prediction. Bioinformatics.

[69] Amiryousefi A, Hyvönen J, Poczai P (2018). IRscope: an online program to visualize the junction sites of chloroplast genomes. Bioinformatics.

[70] Frazer KA, Pachter L, Poliakov A, Rubin EM, Dubchak I (2004). VISTA: computational tools for comparative genomics. Nucleic Acids Research..

[71] Darling ACE, Mau B, Blattner FR, Perna NT (2004). Mauve: multiple alignment of conserved genomic sequence with rearrangements. Genome Res.

[72] Posada D (2008). jModelTest: phylogenetic model averaging. Mol Biol Evol.

